# Reinventing gut health: leveraging dietary bioactive compounds for the prevention and treatment of diseases

**DOI:** 10.3389/fnut.2024.1491821

**Published:** 2024-10-22

**Authors:** Qiurong Wang, Hui Huang, Ying Yang, Xianglan Yang, Xuemei Li, Wei Zhong, Biao Wen, Feng He, Jun Li

**Affiliations:** ^1^Chengdu Medical College, Chengdu, China; ^2^Department of Gastroenterology, The First Affiliated Hospital of Chengdu Medical College, Chengdu, China; ^3^Pengzhou Branch of the First Affiliated Hospital of Chengdu Medical College, Pengzhou Second People’s Hospital, Chengdu, China

**Keywords:** gut microbiota, bioactive compounds, dietary modulation, probiotic, short-chain fatty acid, gut health

## Abstract

The human gut harbors a complex and diverse microbiota essential for maintaining health. Diet is the most significant modifiable factor influencing gut microbiota composition and function, particularly through bioactive compounds like polyphenols, dietary fibers, and carotenoids found in vegetables, fruits, seafood, coffee, and green tea. These compounds regulate the gut microbiota by promoting beneficial bacteria and suppressing harmful ones, leading to the production of key microbiota-derived metabolites such as short-chain fatty acids, bile acid derivatives, and tryptophan metabolites. These metabolites are crucial for gut homeostasis, influencing gut barrier function, immune responses, energy metabolism, anti-inflammatory processes, lipid digestion, and modulation of gut inflammation. This review outlines the regulatory impact of typical bioactive compounds on the gut microbiota and explores the connection between specific microbiota-derived metabolites and overall health. We discuss how dietary interventions can affect disease development and progression through mechanisms involving these metabolites. We examine the roles of bioactive compounds and their metabolites in the prevention and treatment of diseases including inflammatory bowel disease, colorectal cancer, cardiovascular diseases, obesity, and type 2 diabetes mellitus. This study provides new insights into disease prevention and underscores the potential of dietary modulation of the gut microbiota as a strategy for improving health.

## 1 Introduction

The human gastrointestinal tract harbors a vast and intricate community of microorganisms known as the gut microbiota. This dense and diverse ecosystem, comprising bacteria, fungi, viruses, and protozoa, plays a crucial role in maintaining human health (1). The predominant phyla within the gut microbiota include *Bacteroidetes*, *Firmicutes*, *Actinobacteria*, *Proteobacteria*, *Fusobacteria*, and *Verrucomicrobia*, which are considered essential for sustaining overall well-being (2). Research indicates a complex relationship between the gut microbiota and various diseases. Dysbiosis can lead to conditions such as inflammatory bowel disease (IBD) (3), obesity, diabetes (4), and other ailments (5). Although the gut microbiota typically remains stable over time, its composition and abundance can be influenced by numerous factors such as diet, age, geography, lifestyle, and medication use, with diet being the most influential and modifiable factor (6, 7).

Bioactive compounds are non-nutrient constituents of foods that have biological activity in the body, influencing health and disease (8). They encompass a diverse array of chemical substances, including polyphenols, dietary fibers, carotenoids, phytosterols, and alkaloids, which are abundant in plant-based foods like fruits, vegetables, grains, legumes, nuts, and teas. These compounds play a significant role in modulating the gut microbiota composition and activity, which in turn affects host metabolism and immune function (9). For example, bioactive compounds such as polyphenols can directly affect microbial growth by inhibiting bacterial enzymes, disrupting cell walls and membranes, and modulating quorum sensing pathways (10). Catechins can inhibit bacterial DNA gyrase and dihydrofolate reductase, leading to the suppression of pathogenic bacterial growth (11). Bioactive compounds can influence the gut microbiota indirectly by modulating the host’s immune responses. Certain polyphenols can affect immune cell function and cytokine production, which in turn can alter the gut microbial environment (12). For example, quercetin exerts its effects through the modulation of signaling pathways such as NF-κB and mitogen-activated protein kinase (MAPK) in intestinal epithelial cells, leading to decreased pro-inflammatory cytokine production (13). Furthermore, bioactive compounds can serve as substrates for microbial metabolism, leading to the production of beneficial metabolites that influence microbial composition. Dietary fibers, for instance, are fermented by gut bacteria to produce short-chain fatty acids (SCFAs) such as acetate, propionate, and butyrate (14). These SCFAs lower the colonic pH, inhibiting the growth of harmful bacteria and promoting beneficial ones (15). Altered human diets can also induce changes in gut microbiota metabolites levels, with various microbial species producing distinct byproducts such as SCFAs, bile acid derivatives, and tryptophan intermediates. These metabolic byproducts can affect the differentiation and functionality of immune cells via alteration of receptor signalings (16), thereby contributing substantially to the development of disorders such as IBD, malignancy, and a multitude of extraintestinal conditions.

**Figure 1:**
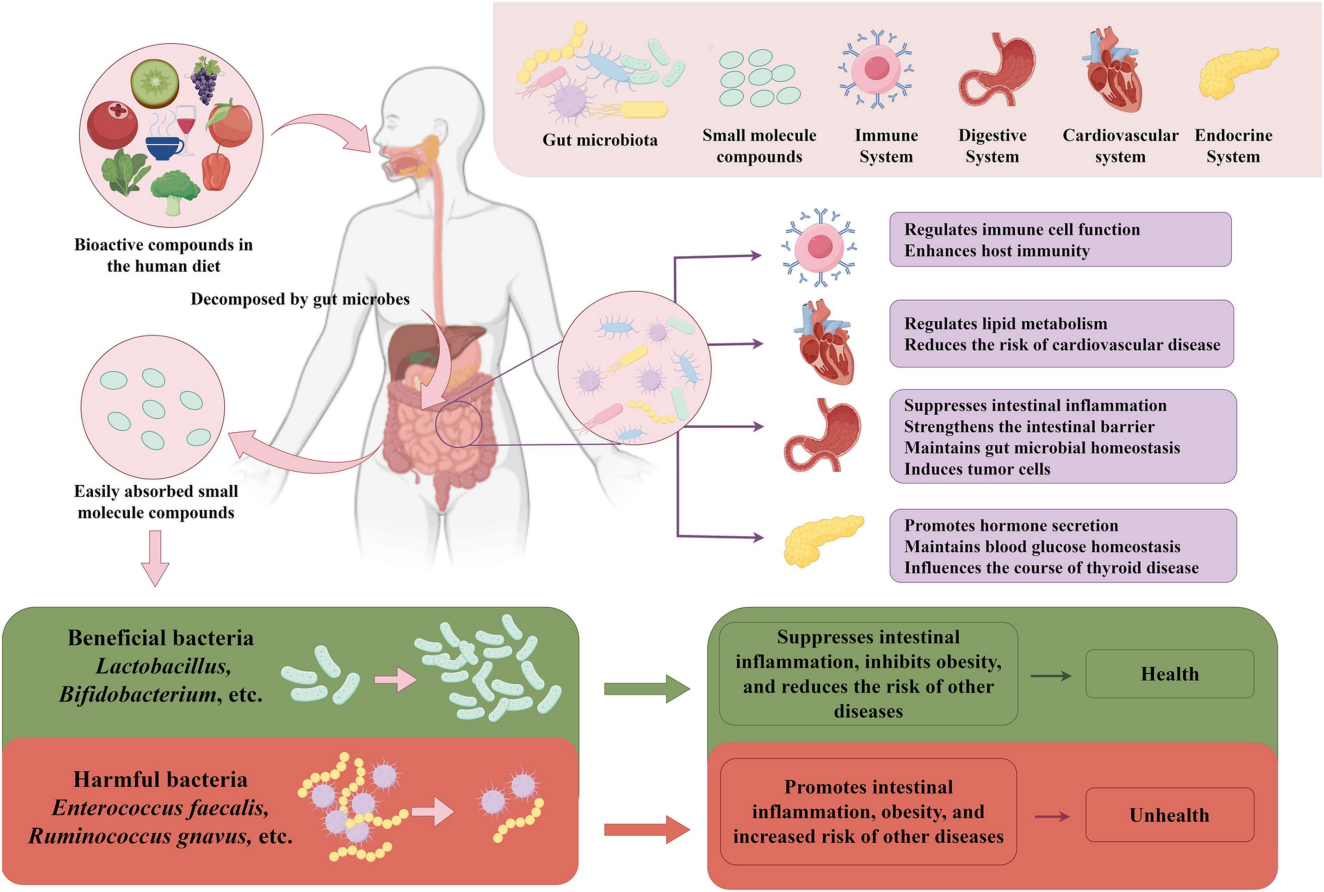
The relationship between gut microbes and bioactive compounds and their impact on host health. Dietary bioactive compounds pass through the intestine, where they are broken down and me-tabolized by enzymes from intestinal microorganisms. This process promotes the growth of beneficial bacteria, such as lactobacilli and Bifidobacterium, and inhibits the proliferation of harmful bacteria, such as *Enterococcus faecalis* and *Ruminococcus gnavus*, thus maintaining intestinal microbial homeostasis. Maintaining gut microbial homeostasis is crucial, as it is closely related to the function of many human systems. For example, gut microbes regulate immune cell function, enhance host immunity; strengthen the intestinal barrier, suppress inflammation; and regulate lipid metabolism, reducing the risk of cardiovascular disease. Additionally, gut microbial home-ostasis promotes hormone secretion, maintains blood glucose homeostasis and influencs thyroid health. The figure was drawn by FigDraw.com.

The present review delves into the influence of bioactive compounds on the constitution and function the gut microbiota, while exploring potential associations linking gut microbiota metabolites to various diseases. We highlight the preventive and therapeutic roles of microbiota-derived metabolites in common diseases and discuss how gut microbiota serves as a mediator for bioactive compound interventions. This investigation offers fresh perspectives on disease prevention and management strategies, emphasizing the potential of dietary modulation to shape the gut microbiota (Figure 1).

## 2 Regulatory effects of bioactive compounds on the gut microbiota

Vegetables, fruits, seafood, coffee and green tea are rich in bioactive elements, demonstrating important physiological functions to maintain their balance of the gut microbiome (Table 1). This section offers a brief summary of their regulatory impacts, which is supported by reliable scientific data.

**Table 1:** Regulation of gut microbes by bioactive compounds.

Biologically active compounds	Gut microbial changes	Experimental model	Reference
Orange juice	Increase the abundance of lactobacilli, *Enterococcus*, *Bifidobacterium*, and *Clostridium*, while reducing the overall abundance of intestinal bacteria.	Human gut microbial ecosystem simulator	(31)
Cocoa	Promote the growth of bifidobacteria and lactobacilli while inhibiting the growth of *Clostridium*.	Human	(39)
Quercetin	Increase the abundance of bifidobacteria, *Bacteroides*, *Clostridium*, and lactobacilli, while reducing the abundance of *Enterococci* and *Fusobacteria*.	Mouse	(42)
Blueberry	Alter the composition of bifidobacteria, lactobacilli *acidophilus*, *Actinobacteria*, *Proteobacteria*, *Dehalobacteria*, *Adlercreutzia*, *Campylobacter, Prevotella*, *Helicobacter pylori*, and *Desulfovibrio* in the gut.	Mouse and human	(50)
Anthocyanin-rich blend of blueberries, black currants and black rice	Enrich the population of *Bacteroidetes* and decrease the abundance of *Firmicutes* and *Actinobacteria*, resulting in a lower *Firmicutes/Bacteroidetes* ratio.	Human	(51)
Grapes	Promote the growth of *Akkermansia*, m*uciniphila* and reduce the *Firmicutes/Bacteroidetes* ratio.	Mouse	(52)
Cranberry extract	Increase the abundance of *Akkermansia*, *Parabacteroides*, and *Barnesiella*, and reduce the abundance of *Bacteroides* and *Prevotella*.	Human gut microbial ecosystem simulator	(53)
Caffeic acid	Decrease the relative abundance of *Bacteroides* and *Turicibacter*, and increase the relative abundance of *Alistipes*, *Akkermansia*, and *Dubosiella*.	Mouse	(62)
Vanillic acid	Increase the *Firmicutes/Bacteroidetes* ratio by boosting the abundance of *Lachnospiraceae, Lachnospira, Eubacterium eligens*, and *Eubacterium*, while decreasing the abundance of *Prevotellaceae*.	Pig	(63)
Resveratrol	Increase the abundance of lactobacilli and bifidobacteria, and increase the *Firmicutes/Proteobacteria* ratio.	Mouse	(64, 65)
Dietary Fiber	The abundance of *Enterococcus faecalis* and *Bifidobacterium adolescentis* significantly increased, while the quantities of harmful bacteria such as *Clostridia*, *Bacteroides*, *Escherichia coli*, and *Eubacterium aerofaciens* decreased.	Human	(83)
Inulin	Increase the abundance of bifidobacteria while reducing the *Firmicutes/Bacteroidetes* ratio.	Rat and mouse	(86)
Oligofructose	Increase the abundance of bifidobacteria in the gut of obese mice.	Mouse	(91)

### 2.1 Polyphenols

Polyphenols are natural compounds with extensive biological activity and potential health-promoting properties. They exhibit antioxidant, anti-inflammatory, antihypertensive, anticancer, and antidiabetic effects (17–19). Most polyphenols in food typically exist in conjugated forms, which makes their molecular structure complex and their bioavailability low. During human digestion, the digestive enzymes in the stomach and small intestine cannot fully break down these polyphenols. As a result, only 5–10% of polyphenols are absorbed in the stomach and small intestine, while the remaining 90–95% enter the colon intact (20, 21). In the colon, polyphenols are metabolized by gut microbiota into monomeric forms of their glycosides or polymeric conjugates. These are then further degraded through reactions such as dehydroxylation, decarboxylation, and aromatic ring cleavage, eventually producing low molecular weight phenolic compounds that can be absorbed by intestinal cells (22). This metabolites influences gut ecology and human health (23). Numerous studies have demonstrated that long-term, moderate polyphenol intake can alter the gut microbiota, fostering a beneficial microbial environment and modifying the *Firmicutes*/*Bacteroidetes* ratio (12, 24), significantly improving human health.

Polyphenols are classified into flavonoids and nonflavonoids on the basis of their chemical composition and complexity (25). Flavonoids, a broadly researched polyphenol group, include diverse compounds such as flavones, flavanones, flavan-3-ols, flavonols, isoflavones, and anthocyanins (26, 27). Nonflavonoids include phenolic acids, particularly hydroxybenzoic acids (e.g., vanillic acid and gallic acid) and hydroxycinnamic acids (e.g., ferulic acid and caffeic acid) (28).

#### 2.1.1 Flavonoid polyphenols

Flavanones are ubiquitous in citrus fruits, including oranges, grapefruits, limes, and lemons (29), with notable concentrations of naringin typically found in grapefruits and hesperidin predominantly in sweet oranges (30). Research has revealed that citrus-derived flavanones possess the capability to hinder detrimental microbial proliferation while promoting the replication of advantageous bacteria such as lactobacilli and *Bifidobacterium*, subsequently modulating the composition of the gut microbiota and preserving intestinal equilibrium (31). At the molecular level, hesperidin has been shown to enhance the expression of tight junction proteins such as occludin and zonula occludens-1 (ZO-1) in intestinal epithelial cells, thereby strengthening the gut barrier function (32). This effect is mediated through the activation of the AMP-activated protein kinase (AMPK) signaling pathway, which also modulates lipid metabolism and reduces inflammation (33). Research by Duda et al. indicated that naringin and hesperidin reduce the populations of *Enterococcus faecalis*, *Bifidobacterium breve*, *Clostridium histolyticum*, *Bacteroides galacturonicus*, and *Escherichia coli* (34). Additionally, incorporating hesperidin into rat diets led to a decrease in abdominal fat and an increase in SCFA production, which was achieved by suppression of pancreatic *α*-amylase activity, thereby increasing colonic microbial fermentation (35). Hesperidin’s influence on SCFA production is also linked to its ability to upregulate the expression of microbial genes involved in carbohydrate metabolism, enhancing fermentation processes (36). After consumption of orange juice for 2 months, the total abundance of anaerobic bacteria and lactobacilli in the stool increased significantly in healthy subjects (37). A growing body of research has demonstrated the beneficial effects of flavanones on gut microbes.

The primary sources of flavan-3-ol monomers (e.g., catechins and epicatechins) are tea, kernel fruits, berries, and cocoa products (38). A randomized controlled trial demonstrated that cocoa flavanols promoted the growth of *Bifidobacterium* and lactobacilli lactis (39). Catechins have been found to inhibit bacterial DNA gyrase and dihydrofolate reductase, essential enzymes for bacterial DNA replication and folate synthesis, respectively, leading to the suppression of pathogenic bacterial growth (11). Moreover, catechins can modulate the host’s gut immune system by influencing dendritic cell function and promoting regulatory T cell differentiation through the modulation of cytokine profiles such as increased interleukin-10 (IL-10) production (40).

Flavonols, such as quercetin and kaempferol, are also phenolic compounds. A previous study revealed that quercetin can reduce the abundance of *Escherichia coli* and *Proteus* species (41). Further research by Shi et al. demonstrated that quercetin could increase the abundance of beneficial *Bifidobacterium*, *Bacteroides*, *Clostridium*, and lactobacilli, while reducing the abundance of *Enterococcus* and *Fusobacterium*, thus maintaining gut homeostasis (42). Quercetin exerts its effects through the modulation of signaling pathways such as NF-κB and mitogen-activated protein kinase (MAPK) in intestinal epithelial cells, leading to decreased pro-inflammatory cytokine production like tumor necrosis factor-alpha (TNF-*α*) and IL-6 (13). Additionally, quercetin can induce apoptosis in pathogenic bacteria by generating reactive oxygen species (ROS) and activating bacterial caspase-like proteins (43).

Anthocyanins, a class of flavonoids, are water-soluble pigments in red wine, cabbage, beans, onions, and berries, contributing to their blue, purple, and red hues (44, 45). Anthocyanin rich blueberries offer significant health benefits, with their polyphenols and various active substances fostering advantageous bacterial proliferation and hindering the replication of harmful bacteria (46). Research has indicated that extracts derived from sea-buckthorns, bilberries, and the dark variety of goji berries promote the proliferation of lactobacilli and *Bifidobacterium* (47–49). Feeding blueberry polyphenol extract to mice revealed that blueberry polyphenol extract affected the quantities of *Bifidobacterium*, *Actinobacteria*, *Helicobacter pylori*, *Deferribacteres*, *Proteobacteria*, *Prevotella*, *Desulfovibrio*, and *Campylobacter* in the gut (50). A clinical study conducted by Vendrame et al. demonstrated that the consumption of blueberries by healthy volunteers led to a rise in *Bifidobacterium* species and lactobacilli *acidophilus* levels (46). Among obese individuals, consumption of a combination of anthocyanins from blueberries, blackcurrants, and black rice led to an increase in *Bacteroidetes* abundance and a reduction in *Firmicutes* and *Actinobacteria* abundance, consequently decreasing the *Firmicutes/Bacteroidetes* ratio (51). This change could help in obesity prevention and treatment. In addition, the intake of grape polyphenols can promote *Akkermansia muciniphila* growth and reduce the *Firmicutes/Bacteroidetes* ratio (52). Cranberry extract increases the abundance of *A. muciniphila* and *Clostridium hironinis* while inhibiting the growth of *Bacteroides* and *Prevotella* species (53). Proanthocyanidins in cranberries can interfere with quorum sensing mechanisms in pathogenic bacteria by inhibiting the autoinducer-2 (AI-2) signaling pathway, reducing virulence factor expression and biofilm formation (54). Red wine, which is also rich in anthocyanins, was shown to alter the dominant fecal bacterial genera from *Bacteroides*, *Clostridium*, and *Propionibacterium* to *Bacteroides*, lactobacilli, and *Bifidobacterium* in rats fed proanthocyanidin-rich red wine extract for 16 weeks (55). Related human studies have shown that consuming red wine and polyphenols significantly increases the abundance of *Enterococci*, *Prevotella*, lactobacilli, *Bifidobacterium*, *Eggerthella lenta*, and *Blautia coccoides*, whereas lactobacilli count remain unchanged (56).

However, it’s important to note that the effects of flavonoids on the gut microbiota are not universally positive. Some studies have reported that high intake of certain flavonoids may disrupt the balance of gut microbiota or have adverse health effects. For instance, excessive consumption of catechins found in green tea has been associated with hepatotoxicity in some individuals. Mazzanti et al. reported cases of liver damage linked to high doses of green tea extracts, suggesting potential toxicity at elevated intake levels (57). Additionally, polyphenols can sometimes inhibit the growth of beneficial gut bacteria. Sánchez-Patán et al. observed that certain polyphenols might suppress beneficial microbial populations while promoting harmful ones, depending on the individual’s gut microbiota composition (58). The metabolism of polyphenols by gut microbiota is highly individualized, and in some cases, metabolites may exert negative effects on health (59). Furthermore, high doses of flavonoids may interfere with the absorption of essential minerals such as iron and zinc, leading to deficiencies (60). Hurrell et al. demonstrated that polyphenol-rich beverages can inhibit non-heme iron absorption in humans (60). These findings indicate that while flavonoids have potential health benefits, their consumption should be balanced, and more research is needed to fully understand their complex interactions with the gut microbiota.

#### 2.1.2 Nonflavonoid polyphenols

Phenolic acids, notably comprising hydroxybenzoic acids (exemplified by vanillic and gallic acids) and hydroxycinnamic acids (including ferulic and caffeic acids), are ubiquitously found in edible produce, including vegetables, fruits, and nuts. Among hydroxycinnamic acids, caffeic acid, ferulic acid, and p-coumaric acid are the most prevalent and pervasively distributed phenolic acids. Studies have shown that ferulic acid can increase the abundance of *Olsenella*, *Eisenbergiella*, *Dubosiella*, *Clostridiales*_*unclassified*, and *Faecalibaculum* in the gut, all of which promote SCFA production (61). Supplementing the diet with caffeic acid can modify the gut microbiota composition by reducing the abundance of *Bacteroides* and *Turicibacter*, while increasing the relative abundance of *Alistipes* and *Dubosiella* (62). Vanillic acid can increase the *Firmicutes*/*Bacteroidetes* ratio, reduce the abundance of *Prevotellaceae*, and increase the abundance of *Lachnospiraceae*, *Lachnospira*, *Eubacterium eligens*, and *Eubacterium*, thereby alleviating gut inflammation in weaned piglets (63). These findings imply that phenolic acids could preserve the diversity and stability of the gut microbiota by promoting the proliferation of advantageous bacteria.

The main representative of stilbenes is resveratrol, which is found primarily in grapes, berries, and peanuts. Research has demonstrated that resveratrol enrichs the populations of lactobacilli and *Bifidobacterium* (64), while also modulating the *Firmicutes* to *Bacteroidetes* ratio in rats (65), thereby modulation the gut microbiota balance, safeguarding gut barrier integrity, and suppressing intestinal inflammation. These changes can also ameliorate obesity and alleviate nonalcoholic fatty liver disease (NAFLD) and nonalcoholic steatohepatitis (NASH) (66, 67). Resveratrol exhibits the capability to diminish body fat accumulation and overall weight through facilitating alterations in the colonic microbiota composition, simultaneously enhancing the management of obesity and glycemic indices, thereby contributing to a more favorable metabolic profile (68).

Dietary lignans are plant hormones that need to be converted into enterolignans by the gut microbiota. The richest source of lignans is oilseeds (69). Research by Corona et al. revealed that lignan-rich oilseeds affect the fecal microbiota of young women and premenopausal women (70).

Despite these potential benefits, there is evidence that some nonflavonoid polyphenols may have adverse effects. Resveratrol, for instance, while showing promise in certain contexts, has also been reported to have low bioavailability and potential pro-oxidant effects at high concentrations, which could lead to cellular damage (71). Moreover, the interaction of resveratrol with the gut microbiota can sometimes result in the production of metabolites that may not be beneficial. Bode et al. found that resveratrol is extensively metabolized by gut microbiota into various metabolites, some of which could have unknown or adverse effects (72). Lignans, although generally considered beneficial, have been suggested to exhibit anti-estrogenic activities, which might interfere with hormonal balance when consumed in high amounts (73). These contradictory findings highlight the need for cautious interpretation of the health effects of nonflavonoid polyphenols and further investigation into their impact on the gut microbiota.

### 2.2 Dietary fiber

Dietary fiber, which consists of plant carbohydrates, is indigestible by human enzymes. Its chemical composition, physicochemical properties and physiological effects vary. Augmenting the amount of dietary fiber in one’s diet can potentially remodel the nutrient-dense environment of the gut, stimulating the growth of intestinal bacteria (74). Only specific gut microbiota constituents can metabolize dietary fiber through anaerobic fermentation, producing SCFAs, including acetate, propionate, and butyrate (14). These metabolites, in turn, influence the configuration, diversity, and abundance of the gut microbiota. Consequently, many research endeavors have focused on elucidating the effect of various dietary patterns on the gut microbiome.

Individuals consuming low-fiber diets often exhibit reduced microbial diversity. Research indicates that incorporating whole grain barley, brown rice, or both into the diets of volunteers enhances microbial diversity (75). For example, investigations have revealed that vegans and vegetarians possess notably greater diversity in their gut microbiota than omnivores (76). At the molecular level, fiber consumption enhances the growth of beneficial bacteria such as bifidobacteria and lactobacilli, which produce bioactive metabolites that regulate inflammation and gut barrier integrity by stimulating the production of mucins and tight junction proteins (77).

Consumption of high-fiber diets is more likely to result in *Prevotella* enterotype dominance in the gut microbiota (78). For example, children from Burkina Faso, who consumed a diet rich in starch, fiber, and plant proteins, had higher *Prevotella* counts than Italian children did (79). Similarly, a comparative study of Indian and Chinese adults revealed that Indians, whose diets contain more plant-based foods, had higher *Prevotella* counts compared to Chinese individuals (80). These results suggest a greater *Prevotella* to *Bacteroides* ratio in individuals consuming high-fiber or vegetarian diets, highlighting the significant impact of dietary habits on the gut microbiota composition.

High-fiber diets also impact the gut microbiota composition by enriching beneficial bacteria and depleting harmful bacteria. The previously mentioned study revealed that children residing in Burkina Faso, who adhered to diets rich in fiber, presented increased abundances of *Bacteroidetes* alongside decreased abundances of *Firmicutes* (79). This decrease in *Firmicutes* abundance has been correlated with efficient obesity prevention and management strategies (81). The molecular mechanisms behind this involve SCFA-mediated activation of peroxisome proliferator-activated receptors (PPARs), which regulate fatty acid storage and glucose metabolism, contributing to reduced fat accumulation and anti-inflammatory effects (82).

Furthermore, an additional study revealed that the consumption of fiber-rich brown rice markedly enriched the fecal populations of *Enterococcus faecalis* and *Bifidobacterium adolescentis*, bacteria known to confer gut health benefits, while simultaneously depleting detrimental microorganisms such as *Clostridia*, *Bacteroides*, *Escherichia coli*, and *Eubacterium aerofaciens* (83). Consequently, consumption of diets high in fiber can modulate the gut microbiota composition by decreasing *Firmicutes* levels, increasing the *Bacteroidetes/Firmicutes* ratio, and inhibiting the proliferation of harmful bacteria, ultimately fostering optimal gut health.

### 2.3 Prebiotics

Prebiotics encompass a diverse array of compounds that remain undigested within the human gastrointestinal tract. These substances are naturally present in a plethora of plant sources, including onion, asparagus, garlic, chicory, ginger, oats, and wheat (84). Common prebiotics include inulin, fructooligosaccharides (FOSs), isomaltooligosaccharides (IMOs), and xylooligosaccharides (XOSs). The consumption of prebiotics fosters the proliferation of advantageous gut microbiota constituents, particularly lactobacilli and *Bifidobacterium* species (85). Inulin, for example, has been shown to enrich bifidobacteria populations and diminish the ratio of Gram-positive bacteria to Gram-negative bacteria (86). At the molecular level, prebiotics like inulin and FOS are fermented by gut microbes into SCFAs, primarily acetate and butyrate, which lower gut pH, inhibiting the growth of pathogenic bacteria (87). Additionally, SCFAs activate gut receptors such as GPR43, which plays a crucial role in regulating immune responses, particularly by reducing pro-inflammatory cytokine production and enhancing the function of regulatory T cells (88).

Some studies have reported a significant increase in the number of bifidobacteria in feces after oligofructose (OFS) consumption (89). Klancic et al. fed OFS to pregnant rats and reported an increase in the abundance of bifidobacteria and *Collinsella* in their offspring, which was associated with a reduced risk of obesity (90). Similarly, Cani et al. demonstrated that OFS increased the number of bifidobacteria in the intestinal tracts of obese mice, contributing to a reduction in adiposity and the inflammatory response (91). Further trials revealed that treating obese women with inulin/OFS prebiotics resulted in increased proportions of bifidobacteria and *E. faecalis* in the body. This effect was consistent with a reduction in fat mass and serum lipopolysaccharide (LPS) levels, indicating a decrease in inflammation (92). This reduction in LPS-driven inflammation is important, as LPS is known to activate Toll-like receptor 4 (TLR4), which triggers pro-inflammatory signaling pathways and insulin resistance (93). These discoveries imply that the consumption of prebiotics is crucial for human wellbeing, as they aid in the restoration of the gut microbiota balance, promote the proliferation of beneficial bacteria, contribute to the prevention and management of obesity, and reduce inflammatory responses.

## 3 Gut microbiota-derived metabolites

**Figure 2:**
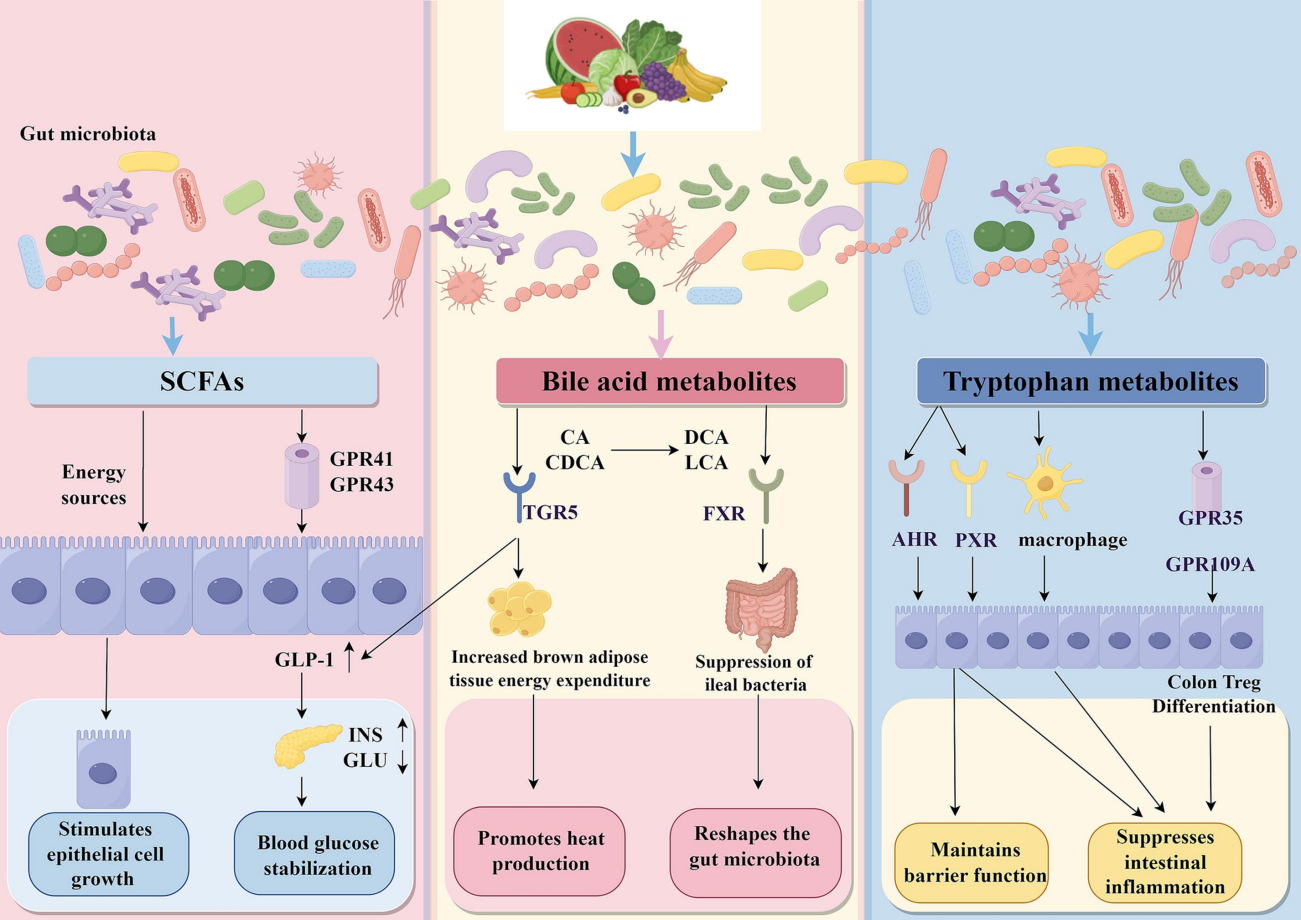
Effects of gut microbial metabolites on host health and their mechanisms. Gut microbial metabolites, including short-chain fatty acids (SCFAs), bile acid metabolites, and tryptophan metabolites, play crucial roles in maintaining host health through various mechanisms. SCFAs, which are produced by the fermentation of dietary fibers, serve as energy sources and stimulate epithelial cell growth. They activate the G protein-coupled receptors (GPR41 and GPR43), enhancing the secretion of glucagon-like peptide-1 (GLP-1), which stabilizes blood glucose (GLU) levels and promotes insulin (INS) secretion. Bile acids, such as cholic acid (CA) and chenodeoxycholic acid (CDCA), are modified by gut bacteria into deoxycholic acid (DCA) and lithocholic acid (LCA). These metabolites interact with the receptors Takeda G protein-coupled receptor 5 (TGR5) and farnesoid X receptor (FXR). Activation of TGR5 increases energy expenditure in brown adipose tissue, while FXR activation suppresses the growth of ileal bacteria and reshapes the gut microbiota. Tryptophan metabolites interact with various receptors, including the aryl hydrocarbon receptor (AHR), pregnane X receptor (PXR), GPR35, and GPR109A, influencing immune responses. They help maintain gut barrier function and suppress intestinal inflammation by promoting the differentiation of regulatory T cells (Treg). The figure was drawn by FigDraw.com.

The gut microbiome produces diverse metabolites critical for gut homeostasis, modulating disease progression via signaling through metabolic and immunological pathways, emphasizing their fundamental role in intestinal balance and disease trajectory (Figure 2). These metabolites, including SCFAs, bile acid derivatives, and tryptophan metabolites, act through specific molecular mechanisms that influence host physiology and immunity.

### 3.1 Short-chain fatty acids

SCFAs are fatty acid compounds with 1–6 carbon atoms (94), produced by the fermentation of dietary fiber by the gut microbiota (95). SCFAs are essential for energy production, lipogenesis, gluconeogenesis, and cholesterol synthesis (96). SCFAs including acetate, propionate, and butyrate, play vital roles in human health by regulating critical biological processes (97). Typically, the molar ratio of acetate: propionate: butyrate is approximately 60:20:20 (98). However, different SCFAs are metabolized by different bacteria, resulting in varying proportions (99). For instance, Bacteroides produce propionate; *Akkermansia*, *Bifidobacterium*, *Prevotella*, and *Bacteroides* produce acetate; and *Faecalibacterium* produces butyrate (100). There is a clear link between SCFA levels and the microbiota composition. The fermentation process increases SCFA concentrations in the gut lumen, lowering the colonic pH and inhibiting the growth of Gram-negative *Enterobacteriaceae* (15). SCFAs, particularly butyrate, serve as beneficial metabolic substrates for energy homeostasis in epithelial cells and are an important source of energy for colonocytes (101, 102). Butyrate stimulates the normal growth and differentiation of epithelial cells, maintains intestinal barrier function, and reduces apoptosis (103, 104).

At the molecular level, SCFAs exert their effects through several mechanisms. SCFAs can enter the bloodstream and activate various G protein-coupled receptors (GPCRs), mainly GPR41 (also known as FFAR3), GPR43 (FFAR2), and GPR109A (HCAR2) (82, 105). Activation of GPR41 and GPR43 on enteroendocrine cells stimulates the release of peptide YY (PYY) and glucagon-like peptide-1 (GLP-1), which regulate appetite and glucose homeostasis (106). GPR109A is specifically activated by butyrate and niacin, affecting lipid, glucose, and cholesterol metabolism (107). For example, acetic acid and propionic acid stimulate the production and secretion of GLP-1 through the activation of GPR43 in the intestinal tract, promoting insulin release and inhibiting glucagon secretion (108). Butyrate independently induces the expression of key intestinal gluconeogenesis genes, such as phosphoenolpyruvate carboxykinase 1 (PCK1) and glucose-6-phosphatase (G6PC), via histone acetylation and activation of the cAMP response element-binding protein (CREB) (109). Additionally, SCFAs protect the gut from infection and inflammation-induced damage through GPR41-mediated promotion of interleukin-22 (IL-22) production by innate lymphoid cells (ILCs) (110).

Moreover, SCFAs inhibit histone deacetylases (HDACs), leading to increased histone acetylation and altered gene expression in immune cells (111). This epigenetic regulation results in anti-inflammatory effects by suppressing the production of pro-inflammatory cytokines such as TNF-*α* and IFN-*γ* (112). Butyrate also promotes the differentiation of regulatory T cells (Tregs) by enhancing the acetylation of the Foxp3 gene promoter, which is essential for Treg development (113). Studies have shown that dietary interventions, such as the addition of legumes to rat diets, can increase SCFA production and the abundance of butyrate-producing bacteria. This increase in butyrate levels has been associated with reduced body weight, lower body fat, and improved insulin sensitivity.

### 3.2 Bile acid metabolites

Bile acids are the end products of cholesterol metabolism and are amphipathic steroid molecules, including primary and secondary bile acids, which are involved in the digestion and absorption of dietary fats and fat-soluble vitamins (114). Primary bile acids, such as cholic acid (CA) and chenodeoxycholic acid (CDCA), are synthesized from cholesterol. Once transported to the intestine, the gut microbiota converts primary bile acids into secondary bile acids through deconjugation and 7-dehydroxylation, resulting in the production of compounds such as deoxycholic acid (DCA) and lithocholic acid (LCA) (115, 116). In humans, bile acids are converted in four different ways, including deconjugation, dehydroxylation, oxidation, and differential isomerization (117), although the specific intestinal bacteria responsible for the production of bile acid metabolites have not been fully elucidated.

The intestinal microbiome governs the constitution of bile acids, subsequently exerting a substantial effect on the configuration of the human gut microbiota. These bile acids, which possess detergent-like characteristics, are capable of disrupting bacterial cellular structures and remodeling microbial assemblages by constraining the proliferation or persistence of specific bacterial taxa. For example, investigations have demonstrated that increased bile acid concentrations in mice promote the proliferation of potential pathogens, such as *Bilophila* and *Desulfovibrio*, concurrently diminishing the abundance of beneficial bacteria such as *Ruminococcus*, lactobacilli, and *Roseburia* (118). Interestingly, Gram-positive bacteria are more sensitive to bile acids compared to Gram-negative bacteria, with bile acids favoring the growth of Gram-negative bacteria (115, 119).

Furthermore, bile acids act as signaling molecules by activating specific receptors, thereby regulating metabolism and immune responses. Bile acid signaling cascades encompass an array of receptors, namely the nuclear hormone receptor farnesoid X receptor (FXR), the G protein-coupled bile acid receptor TGR5 (also known as GPBAR1), and the pregnane X receptor (PXR), each contributing to the intricate regulatory network governing bile acid homeostasis (120, 121). FXR is distributed in various tissues, including the liver and intestines. Upon activation by bile acids, FXR regulates the expression of genes involved in bile acid synthesis, lipid metabolism, and glucose homeostasis (122). In the intestine, FXR activation induces the expression of fibroblast growth factor 19 (FGF19 in humans, FGF15 in mice), which acts in an endocrine manner to suppress hepatic bile acid synthesis via the small heterodimer partner (SHP) pathway (123). FXR also enhances the intestinal barrier function by increasing the expression of tight junction proteins and antimicrobial peptides, thereby inhibiting bacterial overgrowth and translocation (124). This interaction helps shape the gut microbiota composition indirectly through FXR signaling. For instance, mice lacking FXR showed disruption of intestinal epithelial barrier function and increased susceptibility to bacterial translocation (125).

Moreover, secondary bile acids such as DCA and LCA can activate FXR and TGR5 receptors, influencing metabolic processes. The G protein-coupled bile acid receptor TGR5 is expressed in immune cells, brown adipose tissue, and muscle (126), and is involved in immune regulation, energy expenditure, and glucose metabolism (127, 128). Activation of TGR5 by bile acids leads to increased intracellular cyclic adenosine monophosphate (cAMP) levels, stimulating energy expenditure and thermogenesis in brown adipose tissue via the type 2 deiodinase (D2)-mediated conversion of thyroxine (T4) to triiodothyronine (T3) (122).

Among these, LCA and its taurine conjugate, taurolithocholic acid (TLCA), are the most potent ligands for TGR5 (129, 130). The activation of TGR5 signaling by bile acids regulates GLP-1 secretion from enteroendocrine L-cells, affecting insulin secretion and blood glucose levels (131). Another study found that bile acid metabolites can increase energy expenditure in brown adipose tissue and generate heat via TGR5-mediated pathways (122). Additionally, bile acids modulate immune responses by interacting with receptors on immune cells. For example, TGR5 activation on macrophages inhibits NF-κB signaling, reducing the production of pro-inflammatory cytokines (132). This immunomodulatory effect contributes to the anti-inflammatory properties of bile acids.

### 3.3 Tryptophan metabolites

Tryptophan is an essential amino acid found in dietary proteins, and is sourced from foods like oats, milk, tuna, and peanuts. The intestinal microbiota engages in tryptophan metabolism, yielding a plethora of metabolites via three distinct routes: (1) direct conversion of tryptophan by gut microorganisms into several molecules, a subset of which function as ligands for the aryl hydrocarbon receptor (AhR), as previously reported (133); (2) the serotonin biosynthesis pathway (134); and (3) the kynurenine metabolic cascade (135). Indole, indole derivatives, and tryptamine are the major tryptophan metabolites in the gut. Notably, however, different bacteria produce different tryptophan metabolites. For instance, *Peptostreptococcus* converts tryptophan to indolepropionic acid (IPA) and indole-3-propionic acid (IPA) (136), whereas *Bacteroides* and *Clostridium* produce skatole from indole-3-acetic acid (IAA) (137). *Clostridium sporogenes* converts tryptophan into IAA and IPA (138, 139), which play critical roles in maintaining gut homeostasis.

Tryptophan metabolites enhance intestinal epithelial barrier function by increasing the expression of genes involved in maintaining epithelial cell structure and function (140). At the molecular level, many tryptophan metabolites act as ligands for AhR, a transcription factor involved in the regulation of immunity and intestinal barrier function (141). For example, research has shown that IPA activates AhR, promoting the differentiation of goblet cells and mucus production, thereby strengthening the intestinal epithelial barrier and reducing inflammation (139). IPA activates PXR receptors to inhibit intestinal inflammation and enhance intestinal barrier function (142). Activation of PXR by IPA leads to the suppression of NF-κB signaling and downregulation of pro-inflammatory cytokines such as IL-6 and TNF-*α* (143). The anti-inflammatory, barrier-maintaining effects of IPA are mediated by the activation of either PXR or AhR. AhR senses a variety of intestinal signals that are critical for immune responses at barrier sites (141, 144).

PXR plays a crucial role in intestinal inflammation and neoplasia, indicating that metabolites derived from tryptophan regulate the development of colitis and colorectal cancer (CRC). AhR activation increases the production of IL-22 by innate lymphoid cells and Th17 cells, which promotes epithelial regeneration and enhances antimicrobial defense, reducing colitis susceptibility (139). *In vitro* studies have shown that tryptophan metabolites can also achieve anti-inflammatory effects by inhibiting histamine production in macrophages (145). Additionally, some tryptophan metabolites (e.g., nicotinic acid) can act as GPR35 and GPR109A agonists to induce colonic Treg differentiation (146). These G protein-coupled receptors, when activated, promote the expansion of regulatory T cells and the production of anti-inflammatory cytokines like IL-10, which are critical for limiting intestinal inflammation (147). However, more research is needed to fully elucidate the molecular mechanisms underlying the effects of tryptophan metabolites.

## 4 The role of gut microbiota metabolites and bioactive compounds in diseases

The gut microbiota is vital for human health, with microbiota dysbiosis linked to chronic diseases such as IBD, type 2 diabetes mellitus (T2DM), CRC, and cardiovascular diseases (CVDs) (148). Diet and environmental factors significantly influence the composition and function, of the gut microbiota, suggesting potential therapeutic strategies for disease modulation.

### 4.1 Inflammatory bowel disease

IBD, an umbrella term including ulcerative colitis (UC) and Crohn’s disease (CD), represents a protracted gastrointestinal malady characterized by an exaggerated immunological reaction toward the intestinal microbiota. This hyperactive immune response results in relentless inflammatory processes and disrupts the delicate microbial equilibrium within the gut (149). The gut microbiota in IBD patients shows a notable depletion of *Firmicutes* and an increase in *Enterobacteriaceae* and *Proteobacteria*. Various dietary bioactive compounds and their metabolites significantly influence the gut microbiota, exhibiting potential anti-inflammatory effects (9). Nonetheless, the heterogeneity of study designs and patient populations necessitates cautious interpretation of these findings.

Green tea, which is rich in polyphenols, has demonstrated anti-inflammatory properties. Studies in mice have shown that green tea consumption increases the abundance of beneficial bacteria such as *Akkermansia* and lactobacilli, while reducing the abundance of potential pathogens such as *Turicibacter* and *Romboutsia* (150). Additionally, green tea polyphenols downregulate the TLR4/MyD88/NF-κB inflammatory pathway, exerting an anti-inflammatory effect. However, these results are primarily from animal studies, and human clinical trials are limited. Factors such as bioavailability and individual differences in gut microbiota composition may affect the efficacy of green tea polyphenols in humans (57, 58).

Berries, especially blueberries, are rich in anthocyanins and have shown promise in reducing inflammation in colitis models. Supplementation with blueberry extract in colitis-afflicted mice has been shown to reduce the disease activity index (DAI) (151). Treating experimentally induced colitis in mice with blueberry anthocyanin extract has been shown to promote the production of IL-10 and decrease the levels of nitric oxide (NO), myeloperoxidase (MPO), IFN-*γ*, IL-12, and TNF-*α*, indicating the potential anti-inflammatory properties of berry compounds (152). However, variations in study methodologies, such as differences in anthocyanin doses, extraction methods, and animal models, can lead to inconsistent results. Some studies have not observed significant effects, suggesting that more standardized research is needed (153).

Additionally, specific gut microbial metabolites significantly impact the progression of IBD. SCFAs inhibit the activity of NF-κB and HDACs, reducing the levels of proinflammatory cytokines and thereby exerting anti-inflammatory effects (112, 154). Furthermore, SCFAs, especially butyrate, can enhance anti-inflammatory effects by inhibiting HDACs and further suppressing the production of proinflammatory cytokines such as IL-12 (155). Among SCFAs, butyrate is particularly notable for its ability to strengthen the intestinal barrier and modulate immune responses (103, 156). However, the effectiveness of SCFAs may vary depending on individual microbiota composition and the site of inflammation. It is noteworthy that a reduction in the abundance of SCFA-producing bacteria in the gut can adversely affect IBD. A study by Vernia et al. revealed that oral administration of butyrate can increase the efficacy of mesalamine in treating active UC, indicating that a decrease in butyrate-producing bacteria is detrimental to alleviating intestinal inflammation in patients with UC (157). Conversely, some patients may not respond to butyrate supplementation, possibly due to differences in gut microbiota or disease pathology (158).

The bile acid receptor FXR is related to IBD, and FXR agonists can alleviate DSS-induced colitis (125). Research has shown that, compared with healthy individuals, IBD patients have significantly lower serum tryptophan levels, with CD patients having even lower levels than UC patients do (159). Plasma tryptophan concentrations are negatively correlated with the severity of IBD. In both mouse models and IBD patients, dietary tryptophan deficiency exacerbates colitis (160). Therefore, dysregulated tryptophan metabolism in IBD patients accelerates disease progression. However, the exact mechanisms and therapeutic potential of modulating tryptophan metabolism require further investigation.

Currently, strategies for treating IBD using bioactive compounds have made some progress in clinical practice. For example, curcumin, a bioactive ingredient, has been found in studies on IBD patients to significantly reduce intestinal inflammation through the inhibition of the NF-κB signaling pathway and the activity of p38 mitogen-activated protein kinase (MAPK) (161). Additionally, the combination of curcumin with traditional medications has shown efficacy in pediatric IBD patients, with no significant clinical side effects reported (162). Furthermore, supplementing with probiotics such as Bifidobacterium and lactobacilli can modulate gut immunity and reduce intestinal inflammation, providing health benefits (163). However, there is still a lack of sufficient clinical evidence on the efficacy of probiotics in IBD patients. Future efforts should focus on translating findings from animal studies into clinical applications to offer more effective treatment options for IBD patients.

### 4.2 Colorectal cancer

CRC ranks among the most lethal diseases globally and has a high mortality rate. Its incidence increases rapidly with age, although the reasons for this increase remain unclear (153). Numerous risk factors contribute to CRC, including age, IBD, obesity, smoking, dietary habits, and genetics (158). Current treatments for CRC are inadequate because of their limited efficacy, their potential side effects, the development of resistance to chemotherapy, and disease recurrence. However, accumulating research indicates that dietary bioactive compounds and gut metabolites can alleviate CRC (164).

Polyphenol metabolites play a critical role in CRC management by regulating the gut microbiota and directly limiting the growth and proliferation of CRC cells (10). Studies have shown that administering a flavonoid mixture of apigenin and Epigallocatechin-3-gallate (EGCG) to CRC patients reduces cancer recurrence rates (165). However, these findings are based on small-scale studies, and larger clinical trials are necessary to validate the efficacy of these compounds. Moreover, some studies suggest that high doses of EGCG may have pro-oxidant effects and could potentially promote carcinogenesis under certain conditions (71, 166). Additionally, green tea consumption decreases the abundance of *Fusobacterium*, a bacterium strongly associated with CRC (166). The catechin EGCG in green tea induces apoptosis and cell cycle arrest in colon cancer HCT-116 cells, demonstrating its anticancer potential (167). Furthermore, *in vitro* research suggests that green tea polyphenol metabolites inhibit the proliferation of HCT-116 cells (168), indicating that these metabolites may exert anticancer effects, although further research is needed to elucidate the underlying mechanisms. Nevertheless, the bioavailability of polyphenols is limited, and their metabolic transformation by gut microbiota can produce metabolites with different activities, complicating their therapeutic use.

SCFAs, such as propionate, butyrate, and acetate, are produced by the fermentation of dietary fiber by the gut microbiota. A diet low in dietary fiber is associated with a higher risk of CRC development (169). The antitumor effects of dietary fiber are closely linked to these SCFAs. Butyrate, in particular, influences CRC progression through various mechanisms. It accelerates histone acetylation, promotes the proliferation of normal epithelial cells, and promotes CRC cell death while inhibiting their proliferation (170). Additionally, butyrate impedes CRC cell angiogenesis, metastasis, and survival by suppressing Sp1 transcription activation (171). It also induces apoptosis by inhibiting the Wnt/*β*-catenin signaling pathway (172) and enhances anticancer treatment efficacy by modulating cytotoxic CD8+ T cell immunity (173). However, some studies have suggested that butyrate might promote cancer cell survival under hypoxic conditions, indicating a potential dual role depending on the tumor microenvironment (167, 168). Thus, butyrate, as a gut microbial metabolite, plays a pivotal role in preventing CRC development. Comparatively, butyrate appears to have stronger anticancer effects among SCFAs, but the context of its action is important. In addition, propionate plays an important role in CRC. Propionate can induce histone acetylation to inhibit the proliferation of colon cancer cells (174). Luu et al. reported that propionate and butyrate can exert antitumor effects by promoting the expression of genes associated with Tc17 cells and CD8+ cytotoxic T lymphocytes (CTLs) (175).

Bile acid derivatives, including ursodeoxycholic acid (UDCA) and lithocholic acid (LCA), possess the capability to stimulate TGR5 activity, thereby exhibiting anti-inflammatory activity via the augmentation of signaling pathways and the subsequent suppression of proinflammatory cytokine generation, which is mediated by the Toll-like receptor 4 (TLR4) signaling cascade (176). However, secondary bile acids like deoxycholic acid (DCA) have been implicated in promoting CRC by inducing DNA damage and inflammation (177). This suggests that bile acids can have both protective and harmful effects, depending on their specific forms and concentrations. Megna et al. reported that indole-3-methanol (I3C) promotes the expression of the tumor suppressor protein p53 and activates apoptotic factors, leading to apoptosis in colon cancer cells (178). Indoleamine 2,3-dioxygenase 1 (IDO1) is a key enzyme initiating the kynurenine pathway. It has been shown that kynurenine reduces tumor-infiltrating CD8+ cells and mediates the immune evasion of tumor cells (179). Therefore, modulating tryptophan metabolism via the kynurenine pathway could be considered in the future for the treatment of colorectal cancer.

Harmful microbiota-derived metabolites, such as trimethylamine N-oxide (TMAO) and secondary bile acids, can induce tumor formation by damaging DNA and abnormally activating intracellular oncogenic signaling pathways. For example, secondary bile acids promote the PKC-p38 MAPK signaling pathway, accelerating the progression of colorectal cancer (CRC) (177). Additionally, a case–control study demonstrated a positive correlation between TMAO levels and CRC risk (180), though direct evidence confirming TMAO’s role in promoting CRC is still lacking. Some studies have not found a significant association between TMAO levels and CRC risk, indicating conflicting results (180). Further research is needed to clarify its carcinogenic mechanisms.

Currently, the clinical application of bioactive compounds and metabolites in CRC treatment is limited. Some phytochemicals, such as curcumin and resveratrol, have been investigated in clinical trials for their anticancer properties, but results have been mixed due to poor bioavailability and variability in patient responses (164). Prebiotics and probiotics have been proposed as adjunct therapies to modulate the gut microbiota and reduce CRC risk. However, clinical evidence is still emerging, and more large-scale, well-designed studies are needed to establish their efficacy and safety (163). Future therapies may involve personalized approaches targeting specific microbiota compositions and metabolic profiles. The use of microbiota-derived metabolites as biomarkers for CRC risk and as therapeutic agents is a promising area of research. Advances in drug delivery systems may enhance the bioavailability of bioactive compounds, improving their clinical utility.

### 4.3 Cardiovascular diseases

CVDs, including hypertension, atherosclerosis, and heart failure, have high mortality rates globally. Accumulating research indicates that the gut microbiota and its metabolites significantly influence the development and progression of CVD. Dietary interventions can induce beneficial changes in gut microbiota, thereby preventing CVD (181). For example, a diet rich in plant-based products have been shown to lower oxidized low-density lipoprotein cholesterol levels in patients with ischemic heart disease. This dietary pattern also alters the relative abundance of specific gut bacteria, particularly those in the *Ruminococcaceae* and *Barnesiella genera*, and their metabolites, reducing the risk of CVDs (182). Resveratrol exerts a cardioprotective effect by inhibiting the TLR4/NF-κB signaling pathway in rats, thereby reducing the risk of cardiovascular disease (183). In addition, Hobbs and colleagues conducted a study on patients with hypercholesterolemia using supplements such as Omega-3 fatty acids, resveratrol, and flavonoids. The results showed a reduction in both total cholesterol and low-density lipoprotein (LDL) levels, which is of great significance for the prevention and treatment of cardiovascular diseases (184). Among bioactive compounds, resveratrol appears to have strong effects, but its low bioavailability limits its therapeutic potential. Conflicting evidence exists regarding the efficacy of polyphenol supplements in reducing cardiovascular risk, with some studies showing minimal benefits (185). Methodological limitations include small sample sizes, short intervention periods, and variations in study populations.

SCFAs, including propionate, butyrate, and acetate, are crucial gut metabolites that inhibit CVD progression. SCFAs promote the release of the hormones peptide YY (PYY) and GLP-1, which lower blood pressure and inhibit atherosclerosis (186). Propionate, in particular, has been shown to have cardioprotective properties by activating GPR41 signaling in vascular endothelial cells, leading to moderate vasodilation and reduced blood pressure (187). However, the exact mechanisms are not fully understood, and individual variability in gut microbiota composition may influence outcomes.

Current therapies focus on lifestyle modifications and pharmacological interventions. While some supplements like omega-3 fatty acids and flavonoids have been used, the clinical evidence supporting their efficacy is mixed (184). Future prospects include developing therapies that modulate the gut microbiota to enhance the production of beneficial metabolites like SCFAs. Personalized nutrition based on individual microbiota profiles may become a valuable tool in CVD prevention and management.

### 4.4 Obesity

Obesity primarily results from the accumulation of adipose tissue, leading to an inappropriate increase in body weight relative to height. This condition is typically associated with excessive fat accumulation, insulin resistance, and chronic low-grade inflammation (188). Research indicates that, compared with healthy individuals, obese individuals exhibit reduced diversity and richness in their gut microbiota. There is a notable decrease in the abundance of beneficial bacteria such as *Akkermansia muciniphila*, *Faecalibacterium prausnitzii*, and *Bacteroides*, alongside a significant increase in the abundance of *Firmicutes* (189). Gut microbiota plays a role in obesity pathogenesis, but findings are sometimes conflicting (188, 189). Methodological limitations such as differences in diet, genetics, and lifestyle factors make it challenging to establish causality.

SCFAs, which are produced as metabolites by the gut microbiota, regulate the secretion of GLP-1 and PYY via theGPR41 and GPR43 receptors (108). These factors can suppress appetite, increase energy expenditure, and help maintain energy balance, thus preventing obesity (190). Additionally, SCFAs promote the production of thermogenic proteins (PPARγ, PGC1α, and UCP1) and lipid oxidation-related proteins (CPT-I and UCP2), thereby increasing energy expenditure and lipid oxidation to prevent obesity (144). Conversely, a high-fat diet leads to a reduction in SCFA levels (191), whereas a low-fat diet has been shown to increase the abundance of beneficial bacteria such as *Faecalibacterium* and *Blautia*, which are beneficial for lipid metabolism (192). Butyrate and propionate appear to have strong effects in promoting lipid oxidation and energy expenditure (144). However, some studies report that elevated SCFA levels may contribute to increased energy harvest from the diet, potentially promoting obesity (82). This paradox highlights the need for a deeper understanding of SCFA functions and their interactions with host metabolism.

Probiotic and prebiotic interventions have been explored to modulate the gut microbiota in obesity management (192). However, clinical trials have yielded mixed results, and long-term efficacy remains uncertain. Future therapies may focus on personalized approaches, considering individual microbiota compositions to enhance weight loss strategies.

### 4.5 Diabetes

Diabetes is a systemic metabolic disorder characterized by hyperglycemia, arising from either reduced insulin secretion or decreased insulin sensitivity (193). T2DM is the most prevalent form, accounting for 90% of cases, and is characterized by decreased insulin secretion and insulin resistance (194). Increasing evidence suggests that gut microbiota dysbiosis is closely associated with T2DM development. Studies have shown that T2DM patients exhibit reduced gut microbiota diversity (195), with a marked decrease in beneficial bacteria such as bifidobacteria and *Akkermansia*, and an increase in the abundance of harmful bacteria such as *Dallella* (196). Thus, targeting the gut microbiota may present a novel therapeutic approach for treating T2DM and related metabolic disorders.

The Mediterranean diet, which is rich in polyphenols, has been shown to promote the proliferation of beneficial bacteria such as *Bacteroides* and *Clostridium*, thereby assisting in weight management and glycemic control (197). In an animal model of diabetes, a cocoa-rich diet increased the abundance of acetate-producing bacteria, particularly *Blautia*, while reducing the abundance of *Enterococci* and lactobacilli, aligning the gut microbiota composition more closely with that of lean rats (198). Quercetin, a bioactive compound found in plants, has been shown to improve lipid profiles, lower serum glucose levels, increase insulin levels, and reduce oxidative stress in diabetic rats (199). The combined administration of isoquercitrin (a quercetin glucoside) and inulin (a nondigestible polysaccharide) modulates the composition of the colonic microbiota, thereby assisting in the maintenance of glycemic balance and reducing insulin resistance (200). Polyphenols such as quercetin and resveratrol have shown potential in improving insulin sensitivity and glucose metabolism (199). Resveratrol appears to have strong effects, but its clinical efficacy is limited by poor bioavailability (68). Conflicting results exist, with some studies showing no significant benefits (185).

Furthermore, changes in the gut microbiota can lead to alterations in the levels of gut metabolites. Consumption of dietary fiber increases the abundance of SCFA-producing bacteria, thereby increasing gut SCFA levels. This promotes GLP-1 secretion by intestinal cells, which in turn increases insulin levels. SCFAs also improve glycemic control and delay the progression of T2DM by inhibiting NF-κB activation and IκBα degradation, thereby reducing the expression of proinflammatory cytokines (201). In addition to SCFAs, bile acids activate FXR and regulate GLP-1 secretion (131), and the tryptophan metabolite indole also regulates the secretion of GLP-1 (202).

Dietary interventions, including high-fiber diets and polyphenol-rich foods, are recommended for diabetes management (181). However, supplementing specific bioactive compounds has not become standard practice due to inconsistent clinical evidence. Future therapies may involve targeting gut microbiota to enhance SCFA production or using synthetic SCFA analogs.

## 5 Conclusion

This comprehensive review underscores the profound influence of dietary bioactive constituents on the configuration and diversity of the gut microbiota, emphasizing their pivotal role in mitigating and managing an array of health conditions such as IBD, CRC, obesity, and T2DM. However, it is important to maintain a balanced perspective and recognize that our understanding of the intricate interplay between gut microbiota, bioactive compounds, and health is still incomplete. These bioactive agents, including polyphenols, dietary fibers, and carotenoids, which are prevalent in fruits, vegetables, seafood, coffee, and green tea, can reshape the composition and functionality of the gut microbiota, promoting the synthesis of advantageous metabolites such as SCFAs, tryptophan derivatives, and bile acid intermediates. For example, green tea-derived polyphenols have been shown to dampen inflammatory cascades in IBD models by modulating the TLR4/MyD88/NF-κB signaling pathway, and dietary fibers stimulate the production of SCFAs like butyrate, which enhances gut barrier function and modulates immune responses in obesity and diabetes. These metabolites are indispensable for preserving gut homeostasis and modulating disease trajectories via mechanisms encompassing gut barrier reinforcement, immune cell fate specification, and the modulation of anti-inflammatory, antineoplastic, and immunomodulatory activities.

Despite these encouraging discoveries, several unanswered questions remain. For instance, the precise molecular mechanisms by which specific bioactive compounds influence the gut microbiota and subsequent disease progression are not fully elucidated. Additionally, the variability in individual responses to these compounds, possibly due to differences in gut microbiota composition, requires further investigation. Future research should aim to address these gaps by exploring specific questions such as: Which bioactive compounds have the most significant impact on key microbial species associated with health and disease? For example, does quercetin more effectively promote beneficial bacteria like Bifidobacterium compared to other flavonoids? How do individual variations in gut microbiota composition influence the efficacy of these compounds, and can personalized nutrition strategies be developed accordingly? What are the potential synergistic or antagonistic interactions between different bioactive compounds and microbiota-derived metabolites, such as the interaction between polyphenols and prebiotics in enhancing SCFA production?

To investigate these questions in more depth, integrative research methods combining metagenomics, metabolomics, and transcriptomics are best suited. Advanced *in vitro* models like gut-on-a-chip systems can simulate the human intestinal environment, allowing for controlled studies of microbial interactions. Personalized microbiota profiling can help tailor dietary interventions to individual needs. Well-designed clinical trials with larger sample sizes and diverse populations are necessary to validate the therapeutic potential of these compounds and understand their effects across different demographics. By deciphering the intricate interplay among diet, the gut microbiota, and health, we can formulate tailored dietary strategies and interventions geared toward optimizing gut health and effectively preventing or managing diseases. Continued research in this field holds the promise of unlocking new avenues for the prevention and treatment of chronic diseases through dietary modulation of the gut microbiota.
