# Supplementary material for: Reinventing gut health: leveraging dietary bioactive compounds for the prevention and treatment of diseases
Source: Front Nutr. 2024 Oct 22;11:1491821. doi: 10.3389/fnut.2024.1491821 (PMC11534667; doi:10.3389/fnut.2024.1491821)
Supplement: Supplementary file 1 [file Table_1.DOCX]

| **Biologically active compounds** | **Gut microbial changes** | **Experimental model** | **Reference** |
| --- | --- | --- | --- |
| Orange juice | Increase the abundance of *Lactobacilli*, *Enterococcus*, *Bifidobacterium*, and *Clostridium*, while reducing the overall abundance of intestinal bacteria. | Human Gut Microbial Ecosystem Simulator | [28] |
| Cocoa | Promote the growth of *Bifidobacteria* and *Lactobacilli* while inhibiting the growth of *Clostridium*. | Human | [33] |
| Quercetin | Increase the abundance of *Bifidobacteria*, *Bacteroides*, *Clostridium*, and *Lactobacilli*, while reducing the abundance of *Enterococci* and *Fusobacteria*. | Mouse | [36] |
| Blueberry | Alter the composition of *Bifidobacteria*, *Lactobacillus* *acidophilus*, *Actinobacteria*, *Proteobacteria*, *Dehalobacteria*, *Adlercreutzia*, *Campylobacter,* *Prevotella*, *Helicobacter pylori*, and *Desulfovibrio* in the gut. | Mouse and human | [43] |
| Anthocyanin-rich blend of blueberries, black currants and black rice | Enrich the population of *Bacteroidetes* and decrease the abundance of *Firmicutes* and *Actinobacteria*, resulting in a lower *Firmicutes/Bacteroidetes* ratio. | Human | [44] |
| Grapes | Promote the growth of *Akkermansia*, m*uciniphila* and reduce the *Firmicutes/Bacteroidetes* ratio. | Mouse | [45] |
| Cranberry extract | Increase the abundance of *Akkermansia*, *Parabacteroides*, and *Barnesiella*, and reduce the abundance of *Bacteroides* and *Prevotella*. | Human Gut Microbial Ecosystem Simulator | [46] |
| Caffeic acid | Decrease the relative abundance of *Bacteroides* and *Turicibacter*, and increase the relative abundance of *Alistipes*, *Akkermansia*, and *Dubosiella*. | Mouse | [50] |
| Vanillic acid | Increase the *Firmicutes/Bacteroidetes* ratio by boosting the abundance of *Lachnospiraceae, Lachnospira,* *Eubacterium eligens*, and *Eubacterium*, while decreasing the abundance of *Prevotellaceae*. | Pig | [51] |
| Resveratrol | Increase the abundance of *Lactobacilli* and *Bifidobacteria*, and increase the *Firmicutes/Proteobacteria* ratio. | Mouse | [52,53] |
| Dietary Fiber | The abundance of *Enterococcus faecalis* and *Bifidobacterium* *adolescentis* significantly increased, while the quantities of harmful bacteria such as *Clostridia*, *Bacteroides*, *Escherichia coli*, and *Eubacterium aerofaciens* decreased. | Human | [66] |
| Inulin | Increase the abundance of *Bifidobacteria* while reducing the *Firmicutes/Bacteroidetes* ratio. | Rat and mouse | [69] |
| Oligofructose | Increase the abundance of *Bifidobacteria* in the gut of obese mice. | Mouse | [72] |
